# Trends in suicide attempts and suicide deaths before and during the COVID-19 pandemic in New Taipei City, Taiwan: an interrupted time-series analysis

**DOI:** 10.1017/S0033291722001155

**Published:** 2023-07

**Authors:** Yi-Jen Su, Hsiu-Ting Yu, Ting-Yu Liu, Kuan-Hung Lu, Chung-Chieh Tu, Yu-Ching Lin, Ran-Chou Chen

**Affiliations:** 1 Graduate Institute of Behavioral Sciences, Chang Gung University, Taoyuan, Taiwan; 2 Department of Psychiatry, Chang Gung Memorial Hospital at Linkou, Taoyuan, Taiwan; 3 Department of Psychology, National Chengchi University, Taipei, Taiwan; 4 Research Center for Mind, Brain & Learning, National Chengchi University, Taipei, Taiwan; 5 Department of Health, New Taipei City Government, New Taipei City, Taiwan; 6 Department of Biomedical Imaging and Radiological Sciences, National Yang Ming Chiao Tung University, Taipei, Taiwan

**Keywords:** COVID-19, interrupted time-series analysis, suicide attempts, suicide deaths, Taiwan

## Abstract

**Background:**

The coronavirus disease 2019 (COVID-19) pandemic is an unprecedented global health crisis that may cause mental health problems and heighten suicide risk. We investigated the impact of the COVID-19 pandemic on trends in suicide attempts and suicide deaths in New Taipei City, Taiwan.

**Methods:**

The current study used the official daily data on suicide attempts and deaths in New Taipei City, Taiwan (4 million inhabitants) between 2015 and 2020 from the Taiwan National Suicide Prevention Reporting System. Interrupted time-series (ITS) analyses with parameters corrected by the estimated autocorrelations were applied on weekly aggregated data to examine whether the suicide trends during the early COVID-19 pandemic (late January to July 2020) deviated from previous trends (January 2015 to late January 2020). The impact due to the suicide prevention policy change was also examined (since August 2020).

**Results:**

ITS analyses revealed no significant increases in both mean and trend on weekly suicide deaths during the COVID-19 pandemic and after the policy change. In contrast, there was a significant increasing trend in weekly suicide attempts since the COVID-19 outbreak at the rate of 1.54 attempts per week (95% confidence interval 0.49–2.60; *p* = 0.004). Sex difference analysis revealed that, however, this increasing trend was observed only in females not in males.

**Conclusions:**

The COVID-19 pandemic has different impacts on suicides attempts and deaths during the early pandemic in New Taipei City, Taiwan. The COVID-19 outbreak drastically increased the trend of suicide attempts. In contrast, the number of suicide deaths had remained constant in the investigated periods.

## Introduction

The coronavirus disease 2019 (COVID-19) pandemic presents a global public health challenge with serious psychosocial impacts. Nearly all aspects of our lives were greatly affected. The direct and indirect effects of the COVID-19 pandemic and its ‘social distancing’ measures on general mental health can be profound. The associated lockdown, physical distancing, and quarantine resulted in social isolation and loneliness, increasing the risk of mental health difficulties and exacerbate health inequalities (Moreno et al., 2020). Recent meta-analyses suggest that the COVID-19 pandemic led to worsened mental health (e.g. depression, anxiety, and posttraumatic stress) in the general population (Necho, Tsehay, Birkie, Biset, & Tadesse, 2021; Wu et al., 2021) and among healthcare workers (Marvaldi, Mallet, Dubertret, Moro, & Guessoum, 2021; Wu et al., 2021). These findings were corroborated by comparing data before and after the COVID-19 lockdown in the UK, showing increased rates of clinically significant mental distress and mental disorders (Pierce et al., 2020; Shanahan et al., 2022).

### Suicide during the COVID-19 pandemic

Suicide is among the most devastating mental health consequence. There are concerns that suicide rates could increase during the COVID-19 pandemic due to a decline in population mental health (Sher, 2020). Some studies have supported associations of suicide risk (e.g. past-month suicide ideation and attempts) to COVID-19-related distress (Ammerman, Burke, Jacobucci, & McClure, 2021; Caballero-Domínguez, Jiménez-Villamizar, & Campo-Arias, 2022). However, there has been an ongoing debate on the impact of the COVID-19 pandemic on suicide. In the early pandemic, numerous anecdotal reports and news coverages reported a decrease in suicide during the lockdown (Niederkrotenthaler et al., 2020). In contrast, reports of COVID-19-related suicides have been increased worldwide in mass media and literature (Sher, 2020). Evidence suggests the link between previous epidemics (e.g. SARS) and increased risk of suicide (Zortea et al., 2021). Regarding the COVID-19 pandemic, there have been mixed results regarding changes in suicide rates in the early pandemic period. Overall, more studies have found a decrease or no change, and few reported an increase; however, many of them had methodological flaws, such as unreliable sources (e.g. unofficial or secondary sources) and not adjusting for temporal trends (e.g. compared only to equivalent periods of previous years) (Pirkis et al., 2021). Numerous studies that addressed these issues using interrupted time-series (ITS) analyses have reported no such increased risk (Faust et al., 2021; Leske, Kõlves, Crompton, Arensman, & De Leo, 2021; Pirkis et al., 2021; Radeloff et al., 2021; Vandoros, Theodorikakou, Katsadoros, Zafeiropoulou, & Kawachi, 2020). Suicide rates during the first wave of the COVID-19 pandemic were similar to the trend in previous years in Greece (Vandoros et al., 2020), Leipzig, Germany (Radeloff et al., 2021), Queensland, Australia (Leske et al., 2021), and Massachusetts, USA (Faust et al., 2021). Indeed, a recent study across 21 countries found no evidence of a significant increase in suicide rates since the pandemic in any country or region, and 12 countries or areas even showed a decline in suicide rates (Pirkis et al., 2021).

Although studies using rigorous methodology have found no increase in suicide mortality rates in the early COVID-19 pandemic (Faust et al., 2021; Leske et al., 2021; Pirkis et al., 2021; Radeloff et al., 2021; Vandoros et al., 2020), it remains unclear whether suicide attempt rates show a similar pattern. Attempted suicide merits serious consideration, as it is far more common than completed suicide and is the strongest predictor of eventual suicide (Haukka, Suominen, Partonen, & Lönnqvist, 2008). Solid evidence on the association between the COVID-19 pandemic and suicide attempts is relatively scarce. A more recent investigation on Israeli nationally representative sample showed that rates of attempted suicide warranting in-patient hospital treatment significantly dropped during the COVID-19 pandemic (Travis-Lumer, Kodesh, Goldberg, Frangou, & Levine, 2021). However, more studies are required to establish clear empirical generalizations. Also, no studies have examined both trends in suicide attempts and deaths in the time of COVID-19.

### Aims

To fill these gaps, the present study investigated the early impact of the COVID-19 pandemic on rates of both suicide attempts and deaths in Taiwan. Before COVID-19, each year about 3861 people die of suicide in Taiwan, a rate of 16.6 per 100 000 people (men: 22.3; women: 16.6) (Ministry of Health and Welfare, 2021). Taiwan first reported a case of COVID-19 on 22 January 2020 and the first COVID-19 death on 15 February 2020. Given the proximity to (130 km) and extensive linkages with Mainland China, the Taiwan government responded rapidly to the outbreak and effectively managed the pandemic. The number of confirmed COVID-19 cases and deaths in Taiwan have remained very low, with a total of 467 cases and 7 deaths by 31 July 2020 and 799 cases and 7 deaths by 31 December 2020 (Taiwan Centers for Disease Control, 2021). Despite the relatively low COVID-19 incidence, deleterious long-term impacts on mental health were still expected due to fear of infection and widespread social distancing measures implemented across Taiwan. Our main objective was to evaluate the impact of the early COVID-19 pandemic on trends in both suicide attempts and suicide deaths in New Taipei City, Taiwan. ITS analyses with parameters adjusted for autocorrelations and seasonality in data were applied to address the research questions. The present study was exploratory in nature without a priori hypotheses.

## Method

### Study population and data sources

Data for this study are based on residents of New Taipei City, the largest city in Taiwan with 4 million inhabitants. We used official daily data on suicide events (containing attempted and completed suicide) for New Taipei City between January 2015 and December 2020. These data were drawn from the Taiwan National Suicide Prevention Reporting System (NSPRS), a national real-time suicide surveillance system managed by the Taiwan Ministry of Health and Welfare. The NSPRS database is designed to incorporate suicide referrals from multiple information sources (e.g. hospital, police and fire departments, or trained gatekeepers). A complete NSPRS case file contains detailed sociodemographic information (e.g. age, marital status, and employment) and suicide-related information (e.g. suicide manner, history of attempted suicide).

A total of 3034 completed and 38 362 attempted suicide cases were recorded officially during the period of 2015–2020. Of the completed suicide cases, males comprised of the majority of the cases (63.9%) with a mean age of 51.03 (s.d. = 18.25, range: 6–102 years); 36.8% were married and 13.0% were employed. Of the attempted suicide cases, females comprised of the majority of the cases, with an average age of 38.73 (s.d. = 17.35, range: 6–110 years); 30.3% were married and 24.4% were employed. It is noteworthy that information on employment status was unavailable for the majority of completed (60.6%) and attempted suicide cases (31.2%).

The Institutional Review Board of Chang Gung Medical Foundation approved this study (Protocol No. 202001985B0). Data were de-identified and housed in a safe, secure database to ensure confidentiality.

### Statistical analysis

Though existing studies have mostly analyzed data on monthly aggregations, we aggregated daily suicide counts to weekly data to increase the sensitivity in detecting possible changes. The number of suicide deaths and suicide attempts were calculated and analyzed separately.

The ITS analyses were used to examine the impact of the COVID-19 pandemic on suicide in Taiwan. It is a statistical method commonly used for evaluating the effectiveness of interventions which implemented at clearly defined time points. This analysis can examine both mean differences (level) or trend (slope) after a specific time point. We apply this analysis to examine the mean and trend of suicide attempts and deaths at two target time points: the first case of COVID-19 and the reporting policy change in Taiwan by using weekly data of suicide attempts and deaths between 2015 and 2020. The first time of interest is the week 3 of 2020 corresponding to the first COVID case in Taiwan; the second time of interest is week 32 of 2020 corresponding to the new suicide prevention policy change in Taiwan's NSPRS database. According to the newly enacted ‘Suicide Prevention Act’, multiple professionals and personnel can engage in suicide prevention reporting when aware of suicide-related events. As this policy change might substantially increase suicide counts and misled the results, we included this time of interest in the analysis for preventing possible confounding. Two specific time points then divided the study data into three time periods: ‘Before COVID-19’ (2015W01-2020W03); ‘Early COVID-19’ (2020W04-2020W31); and ‘After Policy Change’ (2020W32-2020W52). Specifically, segmented regression analyses were used to evaluate: (a) changes in weekly mean number of suicide attempts/deaths (level) immediately after the COVID-19 pandemic and prevention policy change, and (b) changes in trend of suicide attempts/deaths (slope) before and after the COVID-19 pandemic and prevention policy change. Regression models were fitted to the weekly suicide attempts and death, respectively. Sex differences were also examined and statistically tested in the analyses.

When conducting the ITS analyses, auto-correlations were also estimated for accounting possible seasonality in data (Schaffer, Dobbins, & Pearson, 2021). In order to account for possible seasonality, stepwise autoregressive process with backward search was employed. As seasonality produces autocorrelation at the seasonal lag, with this weekly data the order of 53 autoregressive lags were checked. The backward approach sequentially removed insignificant autoregressive parameters. The maximum likelihood estimates were produced after the order of the model is determined using the Yule–Walker estimation method. The significant auto-correlations were identified and were used to adjust the model parameters. However, the autocorrelation was not considered as the main structure pattern, it was incorporated to account for possible seasonal patterns in the time-series data. Analyses were conducted using the procedure AUTOREG in SAS (version 9.4).

## Results

### Initial summary statistics and plots

Summary statistics of weekly suicide counts during 2015–2020 in New Taipei City, Taiwan are presented in Table 1. The average weekly number of suicide attempts (top panel of Table 1) increased across the three time periods of interest (114.70, 148.14, and 191.29 cases per week). The total sample also showed larger variations during the COVID-19 periods. The lower panel of Table 1 summarizes the statistics for weekly number of suicide deaths. The average weekly number of suicide deaths for the three periods are 9.44, 10.86, and 11.76. The mean demonstrated to be in a slightly increasing trend. The period after the reporting policy change also exhibited larger variations than the other two periods.

**Table 1.** Weekly number of suicide attempts and deaths in New Taipei City, Taiwan before and after the onset of the COVID-19 pandemic, 2015–2020

	Weeks	*M*	s.d.	Range
*Weekly number of suicide attempts*				
Total				
Before COVID-19	263	114.70	20.05	58–180
Early COVID-19	28	148.14	29.45	98–194
After policy change	21	191.29	27.11	149–248
Female				
Before COVID-19	263	75.91	14.76	36–126
Early COVID-19	28	102.07	22.85	61–140
After policy change	21	135.52	20.21	102–179
Male				
Before COVID-19	263	38.78	8.31	17–61
Early COVID-19	28	46.07	9.68	28–64
After policy change	21	55.76	9.89	40–71
*Weekly number of suicide deaths*				
Total				
Before COVID-19	263	9.44	5.90	0–38
Early COVID-19	28	10.86	4.64	2–21
After policy change	21	11.76	7.05	5–37
Female				
Before COVID-19	263	3.39	2.38	0–13
Early COVID-19	28	3.75	2.03	1–9
After policy change	21	4.67	3.25	1–15
Male				
Before COVID-19	263	6.05	4.30	0–25
Early COVID-19	28	7.11	3.42	1–17
After policy change	21	7.10	4.47	1–22

*Note.* ‘Before COVID-19’ period: from 2015W01 to 2020W03; ‘Early COVID-19’ period: from 2020W04 to 2020W31; ‘After Policy Change’ period: from 2020W32 to 2020W52.

**Fig. 1.**
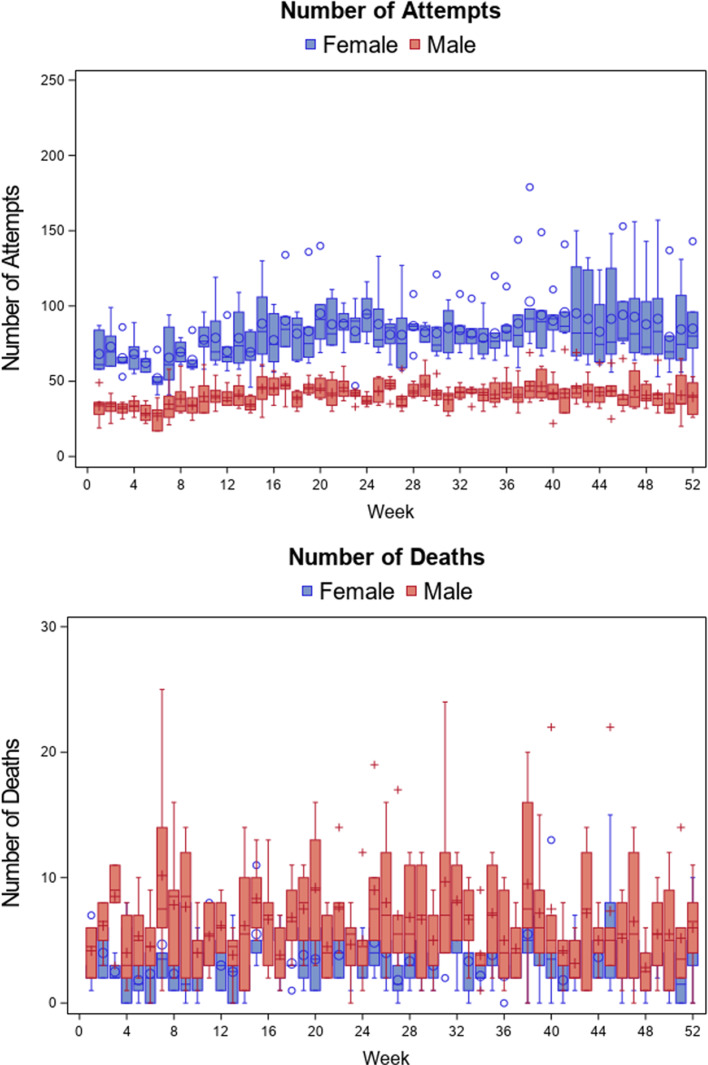
Boxplots of 52 weekly number of suicide attempts and suicide deaths between 2015 and 2020 in New Taipei City, Taiwan.

The boxplots of the 52-week number of suicide attempts and deaths by sex are presented in Fig. 1. The weekly pattern of number of suicides may indicate some seasonal pattern, and thus the seasonal effect was modeled in the following ITS analysis. Figure 1 also clearly shows the sex difference in suicide attempts and deaths. The females had twice the number of suicide attempts than males, but the number of suicide deaths was half of the males.

### ITS analysis

#### Suicide attempts

**Fig. 2.**
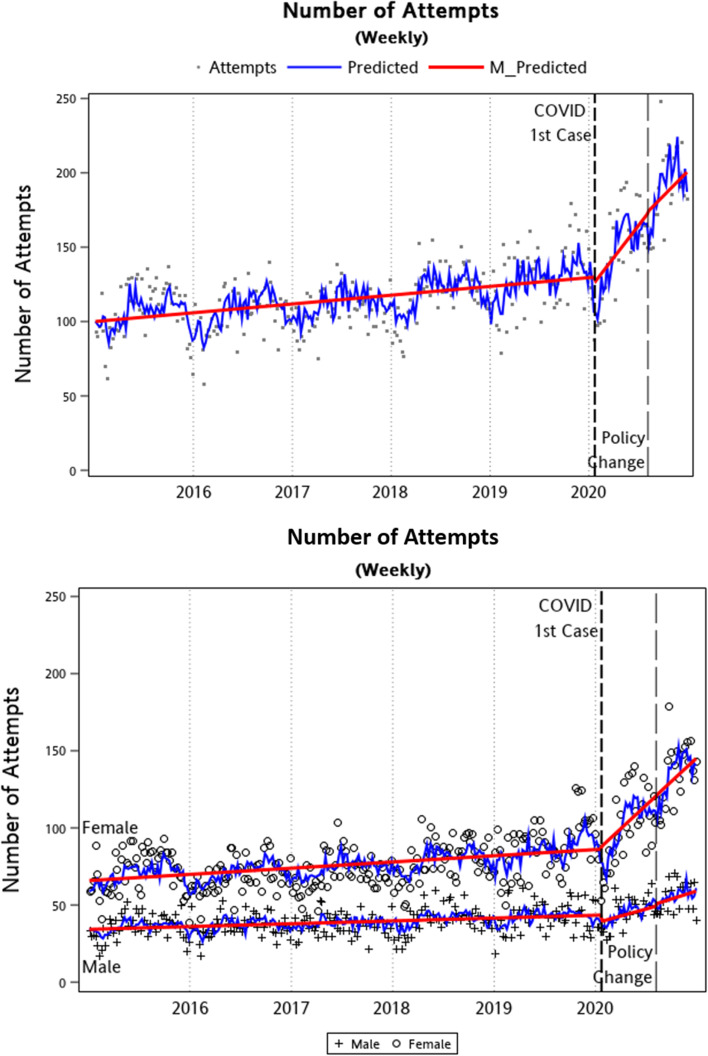
Graphical illustration of ITS analysis – weekly number of suicide attempts between 2015 and 2020 in New Taipei City, Taiwan.

The results of ITS analyses on weekly suicide attempts are summarized in Table 2 and Fig. 2. To account for possible seasonal patterns, the auto-correlations were estimated and tested for significance. For the total sample, number of suicide attempts had a significant weekly increasing trend, with an increasing rate of 0.11 attempts per week during the ‘Before COVID-19’ period (2015W1-2020W3) [95% confidence interval (CI) 0.07–0.16; *p* < 0.001]. Weekly number of suicide attempts was not significantly different after the first COVID-19 case reported on 22 January 2020. However, there was a significant increasing trend during the ‘Early COVID-19’ period (2020W04-2020W31), with an increasing rate of 1.54 attempts per week (95% CI 0.49–2.60; *p* = 0.004). There was no significant impact both on mean and trend in the number of suicide attempts after the change of suicide prevention policy (2020W32-2020W52). The sex difference analyses revealed an overall significant sex difference on mean weekly suicide attempts (see Table 2), i.e. males on average had 31.61 attempts fewer than females. As we used dummy coding (female = 0 and male = 1), when holding all variables constant, results indicated a statistically significant weekly increasing rate of 0.08 on suicide attempts for females during the investigated period (2015 W1 until 2020 W52); while the rate is only 0.04 for males (i.e. 0.08 ± 0.04). Additionally, the estimated rate of the weekly suicide attempts significantly increased for females (rate = 1.09, 95% CI 0.40–1.79; *p* < 0.01), indicating that after the first case of COVID-19, the weekly suicide attempts increase 1.09 case on average per week. However, this increasing trend was observed only in females not in males. Figure 2 graphically presents the results of these analyses discussed above.

**Table 2.** Estimates of coefficients from interrupted time-series analyses: weekly number of suicide attempts and suicide deaths

Variable	Estimate	s.e.	95% CI	
			LL	UL
*Weekly number of suicide attempts*				
Suicide attempts (total)				
Intercept	99.94***	3.45	93.14	106.73
Time (W)	0.11***	0.02	0.07	0.16
Early COVID	−4.25	9.16	−22.27	13.78
Early COVID_WeelyRate	1.54**	0.53	0.49	2.60
PolicyChange	1.34	11.55	−21.38	24.07
PolicyChange_WeelyRate	−0.36	1.06	−2.45	1.73
Suicide attempts (by sex)				
Intercept	65.77***	2.02	61.79	69.74
Sex	−31.61***	2.89	−37.28	−25.93
Time (W)	0.08***	0.01	0.05	0.10
Time (W) × Sex	−0.04*	0.02	−0.08	−0.01
Early COVID	1.17	6.07	−10.75	13.08
Early COVID × Sex	−5.38	8.39	−21.86	11.09
Early COVID_WeelyRate	1.09**	0.35	0.40	1.79
Early COVID_WeelyRate × Sex	−0.78	0.49	−1.74	0.18
PolicyChange	0.21	7.73	−14.96	15.38
PolicyChange × Sex	1.36	10.62	−19.50	22.23
PolicyChange_WeelyRate	0.00	0.70	−1.38	1.38
PolicyChange_WeelyRate × Sex	0.05	0.95	−1.81	1.91

*Weekly number of suicide deaths*				
Suicide deaths (total)				
Intercept	9.14***	0.76	7.63	10.64
Time (W)	0.00	0.00	−0.01	0.01
Early COVID	−1.40	2.15	−5.62	2.83
Early COVID_WeelyRate	0.18	0.12	−0.06	0.42
PolicyChange	0.80	2.97	−5.04	6.65
PolicyChange_WeelyRate	−0.42	0.23	−0.87	0.04
Suicide deaths (by sex)				
Intercept	3.17***	0.37	2.45	3.89
Sex	2.65***	0.52	1.64	3.66
Time (W)	0.00	0.00	0.00	0.01
Time (W) × Sex	0.00	0.00	−0.01	0.01
Early COVID	−0.72	1.25	−3.17	1.73
Early COVID × Sex	0.45	1.76	−3.00	3.91
Early COVID_WeelyRate	0.06	0.07	−0.08	0.20
Early COVID_WeelyRate × Sex	0.02	0.10	−0.18	0.22
PolicyChange	0.49	1.72	−2.89	3.86
PolicyChange × Sex	0.09	2.40	−4.63	4.82
PolicyChange_WeelyRate	−0.12	0.13	−0.38	0.15
PolicyChange_WeelyRate × Sex	−0.14	0.19	−0.51	0.23

*Note.* Sex was dummy coded (female = 0 and male = 1).

*p *<* 0.05, **p *<* 0.01, ***p *<* 0.001.

#### Suicide deaths

**Fig. 3.**
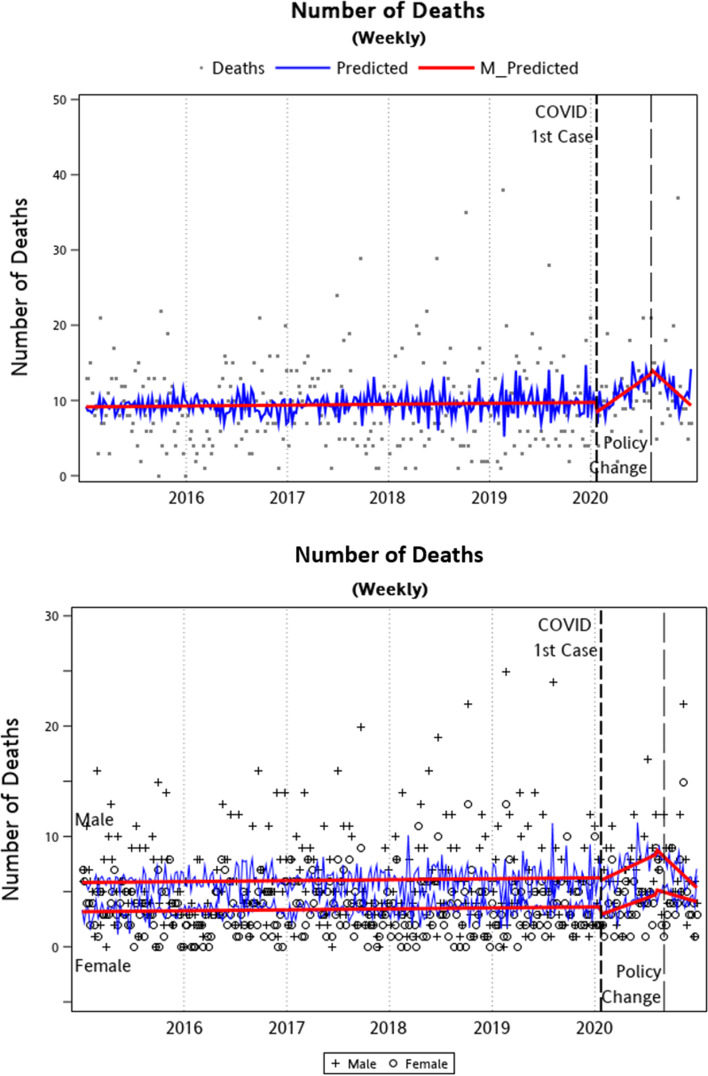
Graphical illustration of ITS analysis – weekly number of suicide deaths between 2015 and 2020 in New Taipei City, Taiwan.

The results of ITS analyses on weekly suicide deaths in New Taipei City are summarized in Table 2 and Fig. 3. Weekly number of suicide deaths revealed no significant changes in both trend and mean during the ‘Early COVID-19’ and ‘After Policy Change’ periods. While the numbers of suicide attempts demonstrated an increasing trend over the past few years, the number of suicide deaths did not show such a trend. With regard to sex differences, there was an overall sex difference on average weekly number of suicide deaths, with males on average having 2.65 more deaths than females (95% CI 1.64–3.66; *p* < 0.001). There were no other sex differences observed after the first case of COVID-19 or policy change. Figure 3 summarizes the analyses graphically for total sample and the male/female subsamples.

## Discussion

This study investigated whether, during the early COVID-19 pandemic, the number of suicide attempts and deaths increased or not in Taiwan. We focused predominantly on the residents of New Taipei City, Taiwan and analyzed data on completed and attempted suicide from 2015 to 2000 using ITS analyses. In general, we found distinct differences in trend of suicide attempts and suicide deaths before and during the COVID-19 pandemic. For the number of suicide attempts, we found a significant increasing trend at the rate of 0.11 cases per week in the suicide attempts before the pandemic, and the rate drastically increased after the first case of COVID-19 at the rate of 1.54 cases per week. However, we did not observe a sudden jump in the mean weekly suicide attempts after the onset of the COVID-19 pandemic.

The results in the number of suicide deaths presented a distinctive picture than the results of suicide attempts. We did not find statistically significant changes in both mean and trend either after the first case of COVID-19 in Taiwan or after a change in suicide prevention policy. The current findings regarding suicide deaths in the early pandemic are consistent with recent studies conducted within single countries/regions (i.e. the USA, Germany, Greece, and Australia) (Faust et al., 2021; Leske et al., 2021; Radeloff et al., 2021; Vandoros et al., 2020) and across multiple countries or regions (Pirkis et al., 2021). Altogether, these findings and ours indicate that the trend in suicide deaths during the early COVID-19 pandemic aligns with the general suicide trends in previous years. Accordingly, there is no compelling evidence that suicide mortality increased during the first wave of COVID-19. A few studies have even found a decreasing trend in the early COVID-19 pandemic (e.g. Pirkis et al., 2021). Despite these results, we cannot completely exclude the possibility of finding an upward trend in some areas, given the heterogeneity in sociocultural contexts and pandemic severity.

There are currently only a few population studies on suicide attempts or self-harm in the time of COVID-19 (Carr et al., 2021; Travis-Lumer et al., 2021). Our results suggest that suicide attempts did show a significant trend change during the early COVID-19 pandemic in the largest city in Taiwan. Though there was a slight increasing trend before the COVID-19 in weekly suicide attempts, the increasing rate drastically intensified since the COVID-19 outbreak. The finding contradicts the observed lower rate of attempted suicide during the COVID-19 relative to pre-COVID years in the Israeli population (Travis-Lumer et al., 2021) and lower incidence of self-harm during the COVID-19 in the UK population (Carr et al., 2021). However, it should be noted that the study in Israel concentrated on severe suicide attempts (i.e. warrants in-patient hospitalization), and the study in the UK identified incident self-harm from UK general practices registered in the database.

There are two main reasons for our finding of an increasing trend of attempted suicide herein. First, our data on suicide attempts included medically serious and non-serious attempts derived from multiple sources (e.g. hospital, police department). Thus, our data were not limited to cases that require emergency department care but also included cases that were reported through other sources (e.g. schools, counseling centers, hot-line call centers). This feature increases the ecological validity and generalizability of the current findings. Second and more important, a national lockdown implemented in the UK and Israel in the early pandemic may limit access to medical services, decreasing the incidence of medically or primary care-recorded attempted suicide, self-harm, and mental illness during the early pandemic (Carr et al., 2021). In contrast, because of the very low COVID-19 incidence, the Taiwan government has maintained multiple effective public health responses (e.g. border closure, mask mandate, strict quarantine measures) without aggressive lockdown measures during the early pandemic. These policies effectively maintained the delivery of routine health services during the pandemic.

We also performed the current analyses in the female and male subsamples. The pattern of results is consistent in both total and sex-stratified samples, but females had a higher increasing rate of suicide attempts than males either before or during the COVID-19 pandemic. Our findings are consistent with recent studies showing that adolescent girls were at greater risk in suicide attempts during the COVID-19 pandemic in the USA (Hill et al., 2021; Yard et al., 2021) and Catalonia, Spain (Gracia et al., 2021). However, those studies conducted a simple comparison of the before-and-after mean rates/numbers of suicide attempts without adjusting for time trends and seasonality (Penfold & Zhang, 2013). Whether these findings still hold with a rigorous statistical analysis (e.g. ITS analysis) is unknown.

### Strengths and limitations

Considering both trends in suicide attempts and suicide death during the COVID-19 pandemic is one of the primary strengths of the present study. This approach helps more fully understand the impact of COVID-19 on population mental health. Moreover, using ITS analyses adjusted for seasonality and temporal trend on weekly suicide data supports the robustness of the findings. The current findings should be considered in light of certain limitations. First, given the lack of comparison cities (controls) in this study, we are uncertain whether our findings apply to the rest of the country. However, New Taipei City is the largest metropolitan area in Taiwan, comprising 17% of Taiwan's total population (4 *v.* 23.5 million). This may improve the generalizability of the study results. Second, the suicide data used may not be sufficiently precise, as some suicide attempts might not be disclosed and registered. Despite this possibility, the Taiwan NSPRS database is well-designed to incorporate all potential information sources, which can probably achieve nationally representative estimates of suicide events in Taiwan population. Finally, we caution the generalizability of our findings to other geographic regions, given Taiwan's unique socio-cultural contexts and successful infection control efforts (i.e. very low COVID-19 incidence).

### Clinical implications

Our findings have major practical implications for public mental health. First, the findings highlight the substantial impact the COVID-19 pandemic has had on population's mental health. The mental problems are caused more by the psychosocial burden of containment measures and/or perceived infection risk than by the severity of the pandemic. Second, rates of attempted suicide and self-harm worldwide during the COVID-19 pandemic are most likely underestimated. Strict lockdown and social distancing measures may have increased barriers to medical emergencies services, which have decreased the number of reported suicide attempts. Despite the finding of an unchanged rate of suicide deaths, we cannot exclude the possibility that the trends in suicide deaths will change due to two reasons. First, there have been few studies showing excess suicide mortality among Japanese (particularly women) in the second half of 2020 (Nomura et al., 2021; Ueda, Nordström, & Matsubayashi, 2021). Second, the mental health effects of the COVID-19 crisis (e.g. depression, anxiety, and insomnia) may take time to emerge and accumulate, particularly for people with mental health histories. Social distancing and lockdown measures could contribute to isolation and loneliness (Groarke et al., 2020), reducing the accessibility to healthcare services and support. The accumulating stress may deplete mental health over time, heightening the later suicide risk. It is crucial to continuously monitor rates of suicide attempts and deaths during the pandemic.

## Conclusions

The present study replicated the recent finding that suicide deaths overall did not increase during the early COVID-19 pandemic. Importantly, however, we found a significant increasing trend of suicide attempts since the COVID-19 outbreak. The present work is valuable not only as a regional but also as a global reference. Further studies are needed to elucidate whether suicide risk during the pandemic increases in vulnerable populations, such as patients with serious mental illness and confirmed COVID-19 patients.
